# Prevalence and Factors Associated With Frailty Among Older Adults Living With HIV Compared to Their Uninfected Peers From the Kenyan Coast

**DOI:** 10.3389/ijph.2024.1606284

**Published:** 2024-02-15

**Authors:** Patrick N. Mwangala, Carophine Nasambu, Ryan G. Wagner, Charles R. Newton, Amina Abubakar

**Affiliations:** ^1^ Institute for Human Development, Aga Khan University, Nairobi, Kenya; ^2^ Centre for Geographic Medicine Research Coast, Kenya Medical Research Institute (KEMRI), Kilifi, Kenya; ^3^ School of Public Health, University of the Witwatersrand, Johannesburg, South Africa; ^4^ MRC/Wits Rural Public Health and Health Transitions Research Unit (Agincourt), Faculty of Health Sciences, University of the Witwatersrand, Johannesburg, South Africa; ^5^ Department of Psychiatry, Warneford Hospital, University of Oxford, Oxford, United Kingdom; ^6^ Department of Public Health, Pwani University, Kilifi, Kenya

**Keywords:** HIV, aging, frailty, correlates, sub-Saharan Africa

## Abstract

**Objectives:** a) To document the prevalence and correlates of frailty among older adults living with HIV (OALWH) and their uninfected peers, and b) Investigate HIV status as an independent predictor of frailty.

**Methods: **This cross-sectional study was conducted between 2020 and 2021 at the Kenyan coast among 440 older adults aged ≥50 years (257 OALWH). Frailty was assessed using the Reported Edmonton Frail Scale. Logistic regression was used to examine the correlates of frailty.

**Results:** The prevalence of frailty was significantly higher among OALWH (24%) than their uninfected peers (13%). HIV seropositivity was not independently associated with frailty. Sleeping difficulties, ageism, higher waist/hip ratio, visiting traditional healers, HIV treatment change/interruption, prolonged illness following HIV diagnosis, and self-reported diabetes were significantly associated with higher odds of frailty. Residing in larger households, having higher income, having friends, being male and light physical activities were significantly associated with reduced odds of frailty.

**Conclusion:** The prevalence of frailty is elevated among OALWH; however, factors other than HIV are predominant, particularly psychosocial factors. Multicomponent interventions are needed to prevent/delay and manage frailty in this setting.

## Introduction

Recent years have witnessed a remarkable rise in the population of older adults in developing countries, including Kenya, making caring for the elderly a public health priority [1]. A better understanding of the factors that influence healthy ageing is crucial for designing appropriate therapies to prevent functional decline, maintain independence, and preserve the quality of life of these adults. In this context, increasing attention has been paid to geriatric syndromes, especially frailty, as a potential explanation of the health diversity among older adults [2]. Although a universal definition is still lacking, frailty is commonly conceptualized as the increased vulnerability to external and internal stressors resulting from a significant loss of physiologic reserve [3]. Several pathophysiological pathways, including cellular senescence, mitochondrial dysfunction, oxidative stress, and dysregulation of inflammatory processes, underlie the frailty syndrome [4]. Growing evidence from systematic reviews also links frailty with adverse health outcomes, including emergency hospital admissions [5], disability [6], poor quality of life [7], dementia [8], and premature mortality [9]. Given the high burden, negative impacts on older adults, and the dynamic nature of frailty, identifying the determinants of frailty is imperative, especially among vulnerable older adults such as those ageing with HIV.

Frailty has been examined extensively in recent years; however, most work has been conducted in older populations in high-income countries (HICs). Some work has been conducted in LMICs, but this has been confined to Brazil, Mexico, China [10] and South Africa [11–14]. So far, results indicate that the prevalence of frailty is highly variable, both between countries and between populations within a country, due to differences in the populations studied and the measurement of frailty. A recent meta-analysis of European studies showed a frailty prevalence of 12% among community-based studies and 45% in non-community-based studies [15]. In Latin America, the prevalence of frailty among community-dwelling older adults was 20%, with a range of 8%–43% in the studies reviewed [16]. Among OALWH, the global prevalence of frailty ranges from 5% to 29% [17]. In SSA, there has been growing attention to the risks of frailty in the ageing population, both in the general population and among people living with HIV (PLWH). To our knowledge, about a dozen studies on frailty have been conducted among older adults in the general population in SSA: South Africa [11–14], Tanzania [18–20], Ghana [13, 14], Nigeria [21], and Burkina Faso [22] with prevalence estimates ranging from 5.4% [11] to 63.3% [21]. Only two studies have focused on OALWH [23], with prevalence estimates ranging from 2.8% to 14.7%.

Identifying the risk and protective factors of frailty may be useful for developing interventions designed to prevent and/or lower the burden that frailty places on a person and provide future directions for public health policy. Indeed, numerous studies have focused on identifying the factors associated with frailty, including the role of biological, lifestyle, and psychological factors [18]. Among older adults in the general population, sociodemographic factors (e.g., being female, older age, low education levels, low income, living alone), physical factors (e.g., obesity, malnourishment, hearing loss, visual impairment, persistent pain), lifestyle factors (e.g., smoking, alcohol consumption, sedentary behaviours) and psychological factors (e.g., depressive symptoms, and sleeping problems) have been identified as risk factors for frailty [24–33]. Most of these studies have focused on sociodemographic factors. However, recently published studies have focused more on lifestyle-related, psychological, and biological factors associated with frailty, which may reflect a growing interest in potentially modifiable factors for frailty. Among OALWH, less research on risk and protective factors has been conducted. In a past systematic review [17], the predictors of frailty included older age, comorbidities, diagnosis of acquired immunodeficiency syndrome (AIDS) and low current CD4^+^ cell count.

Like many parts of SSA, Kenya’s population of older adults is increasing rapidly. In the most recent population census of 2019, the proportion of older adults aged ≥50 years was about 11%, representing approximately 5.2 million individuals [34]. Kenya is also witnessing an increase in chronic age-related conditions [35], which coincides with a high prevalence of HIV among those aged ≥50 years [36]. Hence, establishing the current burden and determinants of frailty is essential if health and social care services are to meet the needs of Kenya’s ageing population. The present study aims to: a) determine the prevalence of frailty among OALWH compared to their uninfected peers; b) investigate HIV status as an independent predictor of frailty in the older adults; and c) investigate the determinants of frailty among the older adults at the coast of Kenya.

## Methods

### Study Design and Setting

This was a cross-sectional study carried out at the Kenyan coast in Mombasa and Kilifi counties between 2020 and 2021. With an estimated population of about 1.5 million people [37], most Kilifi residents are rural inhabitants of the Mijikenda tribe, whose primary source of livelihood is subsistence farming and small-scale trading. In Kilifi, the prevalence of HIV in adults is 4.5% [38]. Mombasa County borders Kilifi to the north and hosts Mombasa City, the second-largest city and chief port of Kenya. It has a population of about 1.2 million residents [37]. Given its urban nature, the county is made up of the local (Mijikenda and Swahili) and immigrant communities from other parts of Kenya. At about 60%, the formal sector provides the majority of employment in Mombasa County [39]. The prevalence of HIV in adults in Mombasa is about 7.5% [38].

### Study Participants and Recruitment

#### Older Adults Living With HIV (OALWH)

We recruited the OALWH from two public HIV-specialized clinics in Mombasa and Kilifi counties (one in each). We specifically selected the two clinics because of their wide client catchment area and their large volume of potential participants. To be included, clients had to be aged ≥50 years of age, have a confirmed HIV seropositivity status, be on HIV treatment, and be willing and able to provide informed consent for their involvement.

In both HIV clinics, two community health volunteers or healthcare providers assisted us in reviewing existing records to identify potential clients. Efforts were made to contact all potential clients who had contact details (in alphabetical order) to invite them to participate in our study. Study introductions were conducted in person by a research assistant before any enrolment. Participant recruitment began in Mombasa County; however, it was interrupted by the onset of the COVID-19 pandemic after recruiting and assessing only 72 OALWH. Upon resumption of project activities, the recruitment and assessment of remaining clients (*n* = 368) took place in Kilifi County.

#### HIV Uninfected Older Adults

All the older adults without HIV were recruited from Kilifi County. The Kilifi Health and Demographic Surveillance System (KHDSS) was used to identify families with eligible older adults. Subsequently, potential participants aged ≥50 years were randomly identified from the existing database and followed up at their homes using Global Positioning System (GPS) coordinates by our trained research assistants. Project information was shared with all individuals who expressed interest in participation. As inclusion criteria, individuals had to be ≥50 years old, inhabitants of Kilifi county, and provide consent to their involvement, including willingness to be tested for HIV using a rapid HIV testing kit (OraQuick) for a confirmation of their HIV seronegative status.

### Sample Size Calculations

We calculated our sample size using a previous study [40], which reported significant differences in the prevalence estimate of frailty between OALWH and their uninfected counterparts. Power analyses in Stata (using effect estimates and comparison of two group proportions) were conducted to estimate the required sample size. An overall sample of 310 was required to detect a difference in frailty between OALWH and uninfected peers at 80% power and a 5% level of statistical significance. A sample of 450 participants was deemed sufficient, allowing for missing data.

### Measures

We programmed all our research instruments on Android tablets using the Research Electronic Data Capture (REDCap) platform [41] for face-to-face interviewer administration. The first author (the study coordinator) trained the research assistants for 2 weeks to facilitate the proper administration of the study tools. All study tools not previously adapted to the local language of Swahili underwent recommended adaptation procedures, that is, forward translation, forward translation review, back translation, harmonization by a panel of experts, pilot testing, pilot testing review, and proofreading [42].

### Sociodemographic and Asset Index Form

Sociodemographic characteristics, including participants’ age, sex, marital status, educational level, occupational status, household size, income, living arrangements and number of dependents, were captured in REDCap. We also collected information on individual and family ownership of disposable assets for asset index computation as a proxy for socioeconomic status. The participants also provided information on their food security in the past week, access to social support, social network of close friends*,* the number of people living with HIV in the household, and whether they were taking care of sick family members at the time and visiting traditional healers.

### General Health Information

We also gathered the participants’ anthropometric details (such as height, weight, blood pressure, waist, and hip circumference), hours spent on sedentary activities in a day, sexual activity, number of medications one was using, self-reported comorbidities, past medical history, and common complaints, e.g., fatigue, pain, sleeping difficulties, visual and hearing problems.

For OALWH, we also asked HIV-specific questions relating to the disclosure of HIV status, access to the HIV clinic, and past medical history, e.g., cART regimen change/interruption and prolonged illness after HIV diagnosis. Information pertaining to their current ART regimen and overall duration on ART were extracted from their medical records. We also collected 10 mL of venous blood samples from the OALWH for viral load measurement.

### Psychosocial Measures

Psychosocial variables included HIV-related stigma, functional disability, loneliness, and age-related discrimination (ageism). All these constructs were assessed using interviewer-administered Likert scales, the brief 12-item HIV stigma scale [43], the 12-item World Health Organization Disability Assessment Schedule 2 [44], UCLA 8-item loneliness scale [45], and the 20-item ageism survey [46]. In each scale, a higher score translates into a greater level of impairment.

### Measures of Frailty

We assessed frailty using the modified Reported Edmonton Frail Scale [47]. It assesses nine domains of frailty: cognition, general health status, functional independence, social support, medication use, nutrition, mood, continence, and self-reported functional performance. Each domain comprises a set of questions examining the construct. Test scores range from 0–18, and participants are classified conventionally into 5 categories, with a higher score representing a higher degree of frailty: 0–5 (not frail), 6–7 (apparently vulnerable), 8–9 (mild frailty), 10–11 (moderate frailty) and 12–18 (severe frailty). In the current study, we collapsed these categories into three: 0–5 (non-frail), 6–7 (pre-frail) and 8–18 (frail) to enable meaningful analysis of the correlates of frailty [48, 49].

### Data Analysis

We conducted all our analyses in STATA version 15.0 (StataCorp LP, College Station, TX, United States). We utilized descriptive statistics to summarize sample characteristics. Specifically, independent Student’s t-test and Chi-square test were used to compare differences in independent variables. Proportions were used to estimate the prevalence of frailty among OALHW and their uninfected peers. To examine HIV status as an independent predictor of frailty, we used logistic regression analyses adjusting for relevant exposure variables that accounted for differences in frailty. Examination of the correlates of frailty applied logistic regression models to explore univariate associations between the binary outcome variables (frailty) and the various exposure variables. Expose variables with a *p*-value <0.15 in the univariate analysis were then entered into the multivariable models using forward selection. In all models, collinearity was checked, and for all hypothesis tests, a two-tailed *p*-value <0.05 was deemed statistically significant. We checked the overall fit of the final models using Hosmer and Lemeshow’s goodness of fit, where a *p*-value of >0.05 was considered a good fit.

## Results

### Sample Characteristics

Our sample comprised 440 participants, 257 (58%) of whom were OALWH. An overall response rate of 90% was achieved at recruitment. Among the respondents, 6 (1.4%) did not complete the outcome measure. Table 1 gives the details of the demographic and biopsychosocial information of these participants. In brief, the mean age of the respondents was 60.1 (SD = 6.9) years and 58.6% were female. The majority of the participants were unemployed (65.5%), had a monthly household income of less than 10,000 Kenyan shillings—about $90 (63.4%), lived in multigenerational households (81.6%), and had caregiving responsibilities (66.4%). Moreover, close to half of them reported being sexually active, and a similar proportion reported that they were not accessing/receiving adequate social support. Adults living with HIV were likely to be younger, unmarried, more educated, have lower monthly household income, live alone, and have fewer dependents.

**TABLE 1:** Characteristics of the study population by HIV status, *n =* 440 (The HIV-associated Neurocognitive Disorders study, Kenya, 2020 and 2021).

Characteristic	Total sample *n* = 440	HIV status		
		HIV uninfected older adults, *n* = 183	HIV-infected older adults, *n* = 257	*p*-value
Age (years)				
50–59	227 (51.6)	84 (45.9)	143 (55.6)	
60–69	171 (38.9)	74 (40.4)	97 (37.7)	0.02
≥70	42 (9.5)	25 (13.7)	17 (6.6)	
Sex				
Female	258 (58.6)	98 (53.6)	160 (62.3)	0.07
Male	182 (41.4)	85 (46.4)	97 (37.7)	
Marital status				
Never married	12 (2.8)	4 (2.2)	8 (3.1)	
Separated/Divorced/Widowed	181 (41.1)	45 (24.6)	136 (52.9)	<0.001^e^
Married/cohabiting	247 (56.1)	134 (73.2)	113 (44.0)	
Education level				
None	162 (36.8)	90 (49.2)	72 (28.0)	
Primary	182 (41.4)	65 (35.5)	117 (45.5)	<0.001^e^
Secondary	73 (16.6)	22 (12.0)	51 (19.9)	
Tertiary	23 (5.2)	6 (3.3)	17 (6.6)	
Employment				
Unemployed	288 (65.5)	126 (68.9)	162 (63.0)	
Employed	116 (26.3)	39 (21.3)	77 (30.0)	0.1
Retired	36 (8.2)	18 (9.8)	18 (7.0)	
Household size				
One to two people	69 (15.7)	15 (8.2)	54 (21.0)	
Three to six people	297 (67.5)	131 (71.6)	166 (64.6)	0.001
Above seven people	74 (16.8)	37 (20.2)	37 (14.4)	
Monthly household income (Ksh)				
≤10,000	279 (63.4)	69 (37.7)	210 (81.7)	
Above 10,000	161 (36.6)	114 (62.3)	47 (18.3)	<0.001
Living arrangements				
Multiple generational families	359 (81.6)	169 (92.3)	190 (73.9)	
Single generational families	41 (9.3)	6 (3.3)	35 (13.6)	<0.001^e^
Alone	40 (9.1)	8 (4.4)	32 (12.5)	
Number of dependents, mean (SD)	3.2 (2.6)	3.6 (2.5)	2.9 (2.7)	0.01
Caring for a sick family member, OM = 2				
Yes	291 (66.4)	104 (57.1)	187 (73.1)	0.001
No	147 (33.6)	78 (42.9)	69 (26.9)	
Social network of close friends (*yes or no question*) OM = 1				
None	19 (4.3)	4 (2.2)	15 (5.9)	
Yes	420 (95.7)	179 (97.8)	241 (91.4)	0.1^e^
Access to instrumental/social support				
None	199 (45.2)	93 (50.8)	106 (41.2)	
Sometimes	215 (48.9)	83 (45.4)	132 (51.4)	0.07
Most of the time	26 (5.9)	7 (3.8)	19 (7.4)	
Seeking the services of traditional healers OM = 4				
No	399 (91.5)	166 (90.7)	233 (92.1)	0.6
Yes	37 (8.5)	17 (9.3)	20 (7.9)	
History of cerebrovascular accident OM = 4				
No	427 (97.9)	178 (97.3)	249 (98.4)	0.5^e^
Yes	9 (2.1)	5 (2.7)	4 (1.6)	
Food insecurity (lack of food in the past week), OM = 3				
Never	293 (67.1)	134 (73.6)	159 (62.4)	
Sometimes	119 (27.2)	45 (24.7)	74 (29.0)	0.002^e^
Most of the time/always	25 (5.7)	3 (1.7)	22 (8.6)	
Asset index score^a^—mean (SD)	2.3 (1.5)	1.9 (1.2)	2.5 (1.6)	<0.001
Body mass index—mean (SD), OM = 11	24.9 (6.0)	24.7 (6.1)	25.0 (5.9)	0.7
High waist-hip ratio				
No	202 (46.3)	77 (42.1)	125 (49.4)	0.1
Yes	234 (53.7)	106 (57.9)	128 (50.6)	
Loneliness score^b^—mean (SD), OM = 3	13.9 (3.7)	13.0 (3.4)	14.6 (3.7)	<0.001
Functional disability score^c^—mean (SD), OM = 2	2.5 (4.3)	1.5 (3.0)	3.1 (4.9)	<0.001
Ageism score^d^—mean (SD)	4.2 (5.9)	3.0 (4.4)	5.0 (6.6)	<0.001
Hours spent in sedentary activities in a day, mean (SD), OM = 12	4.5 (2.6)	4.3 (2.1)	4.6 (2.9)	0.3
Days spent doing light activities in the past week, mean (SD), OM = 5	5.6 (2.4)	4.6 (3.0)	6.4 (1.6)	<0.001
Sexually active, OM = 4				
Yes	206 (47.3)	105 (57.4)	101 (39.9)	<0.001
No	230 (52.7)	78 (42.6)	152 (60.1)	
Sleeping difficulties in the past month, OM = 4				
None	276 (63.3)	125 (68.3)	151 (59.7)	
Sometimes	131 (30.1)	53 (29.0)	78 (30.8)	0.01^e^
Most of the time/always	29 (6.6)	5 (2.7)	24 (9.5)	
Frequent bodily pain				
No	334 (75.9)	134 (73.2)	200 (77.8)	0.3
Yes	106 (24.1)	49 (26.8)	57 (22.2)	
Self-reported hearing ability OM = 4				
Very good	360 (82.6)	151 (82.5)	209 (82.6)	0.90
Averagely/very bad	76 (17.4)	32 (17.5)	44 (17.4)	
Self-reported Hypertension, OM = 4				
* *No	322 (73.9)	138 (75.4)	184 (72.7)	0.5
* *Yes	114 (26.1)	45 (24.6)	69 (27.3)	
Self-reported diabetes, OM = 4				
No	412 (94.5)	175 (95.6)	237 (93.7)	0.4^e^
Yes	24 (5.5)	8 (4.4)	16 (6.3)	
Number of medications participants are currently using, mean (SD), OM = 8	1.6 (1.6)	0.4 (1.2)	2.4 (1.2)	<0.001

All numbers are reported as frequencies with percentages unless otherwise stated *p*-values are for the difference between OALWH and their uninfected peers by sample characteristic.

SD, standard deviation; OM, observation with missing value; Ksh Kenya shillings.

^a^ Score range = 0 to 8, higher scores indicate better socioeconomic status.

^b^ Score range = 8 to 27, higher scores indicate greater loneliness.

^c^ Score range = 0 to 33, higher scores indicate increasing disability.

^d^ Score range = 0 to 34, higher scores indicate increasing agism.

^e^ based on Fisher’s exact test.

### HIV-Related Characteristics of Older Adults Living With HIV

All the OALWH were receiving HIV treatment, most (90%) of whom were on a first-line cART regimen. Most (95.3%) of them had disclosed their HIV status. The mean (SD) duration of HIV treatment was 11.4 (4.3) years. Additionally, nearly all of them (98.1%) had suppressed viral load (≤1,000 copies/mL). Further details are highlighted in Table 2.

**TABLE 2:** HIV-related, clinical, and psychosocial characteristics of OALWH, *n =* 257 (The HIV-associated Neurocognitive Disorders study, Kenya, 2020 and 2021).

Characteristic	Mean (SD) or frequency (%)
HIV status disclosure	
Yes	245 (95.3%)
No	12 (4.7%)
Household HIV burden, mean (SD); OM = 5	1.4 (1.6)
cART regimen	
First line	233 (90.7%)
Second line	23 (8.9%)
Third line	1 (0.4%)
cART regimen change/interruption since HIV diagnosis	
Yes	110 (42.8%)
No	147 (57.2%)
Duration on cART (years), mean (SD), OM = 10	11.4 (4.3)
Viral suppression, OM = 45	
Yes	208 (98.1%)
No	4 (1.9%)
Access to HIV clinic, OM = 4	
Easily accessible	169 (66.8%)
Not easily accessible	84 (33.2%)
History of a neurological condition, OM =4	
No	236 (93.3%)
Yes	17 (6.7%)
History of prolonged illness following HIV diagnosis, OM = 6	
No	223 (88.8%)
Yes	28 (11.2%)
Perceived HIV-stigma score, OM = 1	
Personalized stigma^a^—mean (SD)	5.0 (1.9)
Disclosure concerns^b^—mean (SD)	8.6 (2.0)
Concerns about public attitudes^c^—mean (SD)	7.6 (2.2)
Negative self-image^d^—mean (SD)	6.4 (2.1)
Overall stigma^e^—mean (SD)	27.5 (5.4)

^a^ Score range = 3 to 12, higher scores indicate greater stigma.

^b^ Score range = 3 to 12, higher scores indicate greater stigma.

^c^ Score range = 3 to 12, higher scores indicate greater stigma.

^d^ Score range = 3 to 12, higher scores indicate greater stigma.

^e^ Score range = 12 to 44, higher scores indicate greater stigma; OM, observation with missing value; cART, combination antiretroviral therapy.

### Frailty Prevalence Estimates

The overall prevalence of frailty across the sample was 19.4% (95% CI: 15.7–23.4). Groupwise, older adults living with HIV presented with a significantly higher prevalence of frailty (23.9%) than their uninfected peers (12.8%), *p < 0.01* (Table 3).

**TABLE 3:** Prevalence of frailty in OALWH versus their uninfected peers (The HIV-associated Neurocognitive Disorders study, Kenya, 2020 and 2021).

	Older adults without HIV, *n =* 179		Older adults living with HIV, *n =* 255		Total sample, *n* = 434		*p*-value (between groups)
	Number	Prevalence (95% CI)	Number	Prevalence (95% CI)	Number	Prevalence (95% CI)	
Not frail	119	66.5 (59.1–73.3)	140	54.9 (48.6–61.1)	259	59.7 (54.9–64.3)	0.02
Prefrail	37	20.7 (15.0–27.3)	54	21.2 (16.3–26.7)	91	21.0 (17.2–25.1)	0.9
Frail	23	12.8 (8.3–18.7)	61	23.9 (18.8–29.6)	84	19.4 (15.7–23.4)	<0.01

95% CI, 95% confidence interval.

Note: The number of respondents reported in this table (434) is slightly lower than the overall sample size (440) because six participants were dropped from the analysis due to incomplete data on the frailty tool.

### Association Between HIV Status and Frailty

In univariate logistic regression analyses (Table 4), HIV seropositivity was significantly associated with higher odds of frailty (OR 2.13; 95% CI 1.26, 3.60). However, in the multivariable logistic regression model (Table 4), HIV seropositivity was not significantly associated with frailty (aOR 1.26; 95% CI 0.60, 2.63).

**TABLE 4:** Association between HIV status and frailty among older adults (The HIV-associated Neurocognitive Disorders study, Kenya, 2020 and 2021).

Covariate	Positive screen for frailty	
	Crude analysis OR (95% CI)	Adjusted analysis aOR (95% CI)
HIV status		
Seronegative	Ref	Ref
Seropositive	2.13** (1.26, 3.60)	1.26 (0.60, 2.63)
Sex		
Female		Ref
Male		1.29 (0.62, 2.70)
Age (years)		
50–59		Ref
60–69		1.26 (0.64, 2.50)
Above 70		1.20 (0.40, 3.61)
Abnormal waist-hip ratio		
No		Ref
Yes		2.75** (1.41, 5.37)
Household size		
One to two people		Ref
Three to six people		0.37* (0.17, 0.82)
Seven and above		0.36 (0.12, 1.04)
Monthly household income (Ksh)		
≤10,000		Ref
Above 10,000		0.49 (0.22, 1.08)
Social network of close friends		
No		Ref
Yes		0.34 (0.10, 1.17)
Caring for a sick family member		
No		Ref
Yes		0.51* (0.27, 0.97)
Functional disability score		1.05 (0.98, 1.12)
Ageism score		1.08** (1.03, 1.13)
Sleeping difficulties for the past month		
None		Ref
Sometimes		3.70** (1.93, 7.10)
Most of the time/always		2.94 (0.99, 8.76)
Sexually active		
No		Ref
Yes		0.54 (0.26, 1.10)
Seeking the services of traditional healers		
No		Ref
Yes		2.95* (1.24, 7.05)
Self-reported hearing		
Very good		Ref
Averagely/very bad		1.58 (0.78, 3.22)
History of cerebrovascular accident		
No		Ref
Yes		5.87 (0.85, 40.68)
Number of individuals in the final model		427
Hosmer-Lemeshow Test		X^2^ = 402.62; *p* = 0.36
Variance explained		30.81%

CI, confidence interval; OR, odds ratio; aOR, adjusted odds ratio; Ref, reference group.

* – *p*-value < 0.05, ** – *p*-value < 0.01.

Note: The number of individuals in the final model (427) is slightly lower than the actual sample size (440) because some of the observations had missing values.

### Determinants of Frailty in Older Adults Living With HIV

Table 5 presents results from univariate and multivariate logistic regression analyses exploring the determinants of frailty among older adults living with HIV.

**TABLE 5:** Univariate and multivariable analysis of correlates of frailty among OALWH (The HIV-associated Neurocognitive Disorders study, Kenya, 2020 and 2021).

Covariate	Positive screen for frailty by mod-REFS	
	Univariate analysis OR (95% CI)	Multivariable analysis aOR (95% CI)
Age (years)		
50–59	Ref	Ref
60–69	0.96 (0.53, 1.76)	1.58 (0.63, 3.96)
≥70	0.66 (0.18, 2.41)	0.33 (0.05, 2.05)
Sex		
Female	Ref	Ref
Male	1.09 (0.60, 1.99)	1.32 (0.52, 3.39)
Monthly household income (Ksh)		
≤10,000	Ref	Ref
Above 10,000	0.26*** (0.09, 0.76)	0.13** (0.03, 0.60)
Household size		
One to two	Ref	Ref
Three to six	0.33*** (0.17, 0.63)	0.35** (0.12, 0.99)
Above seven	0.26*** (0.09, 0.73)	0.26 (0.06, 1.15)
Caring for a sick family member		
No	Ref	—
Yes	0.13*** (0.07, 0.24)	—
Having close friends (*social network*)		
No	Ref	Ref
Yes	0.09*** (0.03, 0.31)	0.13*** (0.03, 0.56)
Access to instrumental/social support		
Not accessing	Ref	—
Sometimes	2.53*** (1.33, 4.84)	—
Most of the time/always	1.48 (0.44, 5.05)	—
Food insecurity (lack of food in the past week)		
Never	Ref	
Sometimes	2.36*** (1.27, 4.40)	—
Most of the time/always	1.25 (0.43, 3.67)	—
A+sset index, mean (SD)	1.16* (0.97, 1.38)	—
Waist hip ratio		
Normal	Ref	Ref
Abnormal	2.23*** (1.22, 4.06)	3.67*** (1.48, 9.10)
Self-reported Hypertension		
* *No	Ref	—
Yes	0.37*** (0.19, 0.74)	—
Loneliness score, mean (SD)	1.15*** (1.06, 1.24)	—
Functional disability score, mean (SD)	1.13*** (1.06, 1.20)	—
Ageism score, mean (SD)	1.14*** (1.08, 1.19)	1.11*** (1.04, 1.18)
Days spent doing light activities in the past week, mean (SD)	0.69*** (0.58, 0.82)	0.77*** (0.61, 0.98)
Hours spent in sedentary behaviours in a day, mean (SD)	0.79*** (0.70, 0.90)	—
Sexually active		
No	Ref	—
Yes	0.60* (0.33, 1.11)	—
Sleeping difficulties in the past month		
None	Ref	Ref
Sometimes	5.44*** (2.77, 10.71)	2.80** (1.14, 6.87)
Most of the time/always	7.82*** (3.04, 20.15)	1.92 (0.49, 7.43)
Seeking the services of traditional healers		
No	Ref	Ref
Yes	5.60*** (2.17, 14.46)	9.86*** (2.45, 39.68)
HIV status disclosure (*family/relatives*)		
No	Ref	—
Yes	3.55*** (1.91, 6.61)	—
History of a neurological condition		
No	Ref	—
Yes	5.15*** (1.87, 14.21)	—
Prolonged period of illness following HIV diagnosis		
No	Ref	Ref
Yes	3.72*** (1.66, 8.35)	6.58*** (2.13, 20.33)
ART Regimen		
1st line	Ref	—
2nd line	3.07*** (1.27, 7.38)	—
Mean HIV duration (SD)	1.05* (0.98, 1.13)	—
Viral suppression		
Yes	Ref	—
No	8.56* (0.87, 84.01)	—
cART regimen change/interruption since HIV diagnosis		
No	Ref	Ref
Yes	6.86*** (3.55, 13.26)	3.36*** (1.43, 7.91)
Access to HIV clinic		
Not easily accessible	Ref	—
Easily accessible	1.77* (0.90, 3.52)	—
Perceived HIV-stigma score, mean (SD)		
Personalized stigma	0.85* (0.72, 1.00)	—
Disclosure concerns	1.37*** (1.16, 1.62)	—–
Concerns about public attitudes	1.18** (1.02, 1.36)	—
Overall stigma	1.05** (0.99, 1.11)	—
*n* for the final model		248
Variance explained		43.35%
Hosmer-Lemeshow test		X^2^ = 235.00; *p*-value = 0.07
cvMean AUC (95% CI)		0.90 (0.86, 0.95)

Only *a priori* variables (age, sex) and those with *p*-value < 0.15 in the univariate analysis or multivariable *p* < 0.05 are presented here. OR, odds ratio; aOR, adjusted odds ratio; Ref, reference group; cvMean AUC, cross-validated mean area under the curve for the final multivariable model.

* – *p*-value < 0.15, ** – *p*-value < 0.05, *** – *p*-value < 0.01.

Note: The number of individuals in the final model (248) is slightly lower than the actual sample size for older adults living with HIV (257) because some of the observations had missing values.

In the multivariable logistic regression model, factors significantly associated with higher odds of frailty among OALWH were sleeping difficulties in the past month, increasing ageism scores, visiting traditional healers, high waist-to-hip ratio, a history of cART regimen change/interruption, and a history of prolonged illness after HIV diagnosis. On the other hand, having a social network of close friends, a larger household, a higher household income (≥10,000 Ksh; about $86) and taking part in light physical activities such as walking in the past week were significantly associated with lower odds of frailty.

### Determinants of Frailty in Older Adults Without HIV

In multivariable analyses (Table 6), sleeping difficulties in the past month, self-reported diabetes, and light physical activities in the past week were significantly associated with higher odds of frailty among the HIV uninfected older adults. Conversely, being male was significantly associated with reduced odds of frailty in these adults.

**TABLE 6:** Univariate and multivariable analysis of correlates of frailty among HIV-uninfected older adults (The HIV-associated Neurocognitive Disorders study, Kenya, 2020 and 2021).

Covariate	Positive screen for frailty by mod-REFS	
	Univariate analysis OR (95% CI)	Multivariable analysis aOR (95% CI)
Age (years)		
50–59	Ref	Ref
60–69	1.73 (0.62, 4.81)	0.91 (0.26, 3.22)
≥70	3.38** (1.02, 11.24)	4.61 (0.95, 22.29)
Sex		
Female	Ref	Ref
Male	2.29* (0.89, 5.86)	0.26** (0.07, 0.94)
Monthly household income (Ksh)		
≤10,000	Ref	—
Above 10,000	0.51* (0.21, 1.24)	—
Number of close family members/friends one interacted with at least once in the past month, mean (SD)	0.90* (0.81, 1.01)	—
Loneliness score, mean (SD)	1.11* (0.97, 1.26)	—
Functional disability score, mean (SD)	1.13** (1.00, 1.27)	—
Ageism score, mean (SD)	1.08* (0.99, 1.17)	—
Days spent doing light activities in the past week, mean (SD)	1.25** (1.03, 1.52)	1.29** (1.01, 1.66)
Sexually active		
No	Ref	—
Yes	0.27*** (0.11, 0.70)	—
Sleeping difficulties in the past month		
None	Ref	Ref
Sometimes	6.27*** (2.35, 16.70)	4.30** (1.41, 13.07)
* *Most of the time/always	11.05** (1.58, 77.26)	14.72** (1.11, 194.78)
Self-reported hearing		
Very good	Ref	—
Averagely/very bad	4.69*** (1.83, 11.99)	—
Frequent bodily pain		
No	Ref	—
Yes	2.39* (0.97, 5.89)	—
Self-reported diabetes		
No	Ref	Ref
Yes	8.00*** (1.85, 34.64)	8.35** (1.23, 56.92)
Self-reported hypertension		
No	Ref	—
Yes	3.29** (1.33, 8.11)	—
Number of medications participants are currently using, mean (SD)	1.38* (0.98, 1.93)	—
*n* for the final model		177
Variance explained		30.33%
Hosmer-Lemeshow test		X^2^ = 110.02; *p*-value = 0.97
cvMean AUC (95% CI)		0.87 (0.79, 0.94)

Only *a priori* variables (age, sex) and those with *p*-value < 0.15 in the univariate analysis or multivariable *p* < 0.05 are presented here. OR, odds ratio; aOR, adjusted odds ratio; Ref, reference group; cvMean AUC, cross-validated mean area under the curve for the final multivariable model. * – *p*-value < 0.15, ** – *p*-value < 0.05, *** – *p*-value < 0.01.

Note: The number of individuals in the final model is slightly lower than the actual sample size of older adults without HIV because some of the observations had missing values.

## Discussion

Our study adds to the growing number of reports on the burden and determinants of frailty in low- and middle-income countries. In this study, the prevalence of frailty was significantly higher in OALWH (23.9%; 95% CI 18.8–29.6) compared to their uninfected peers (12.8%; 95% CI 8.3–18.7); however, HIV seropositivity was not significantly associated with frailty after adjusting for biopsychosocial factors. To our knowledge, there have been only two previous studies of frailty among OALWH in SSA. The first one, a cross-sectional sample of 145 OALWH (67% female) on ART and a median age of 57 years in Tanzania, reported a low prevalence of frailty (2.8%) [50]. The other one, a population-based cohort of 614 older adults (292 OALWH) in South Africa, reported a frailty prevalence of 17.7% in OALWH compared to 14.7% in their uninfected peers [51]. The variations in frailty prevalence could partly be attributed to differences in frailty measurement and the fact that older adults are a highly heterogeneous group, having different genetic backgrounds, medical profiles, and biological, as well as social-environmental factors at different stages of life, thus highlighting the need for country-specific frailty data using tools validated within the country or region of interest. Our finding of a higher prevalence of frailty in OALWH than their uninfected peers is consistent with previous evidence, which has demonstrated both higher frequency of frailty in OALWH and the development of frailty at earlier ages for OALWH than for individuals without HIV [17]. Many factors may predispose OALWH to elevated rates of frailty, including the effects of persistent inflammation from HIV (even in well-controlled viraemia), toxic effects of earlier cART regimens, delayed initiation of ART, and higher rates of multimorbidity [52].

Literature suggests that it is a constellation of environmental, disease-specific, and biological factors that contribute to frailty [17, 24–33], though most of the evidence is concentrated in HICs. In the present study, the factors significantly associated with frailty were predominantly psychosocial, many of which are potentially modifiable with appropriate programs and interventions. Consistent with previous studies [33, 53], our study showed that sleeping difficulties were associated with higher odds of frailty in OALWH and their uninfected peers. Sleep problems may impact frailty in numerous ways, including decreased energy expenditure, elevated inflammatory response, disturbed hormonal pathways, tissue growth and repair [53]. These mechanisms may explain the associations observed. Interventions targeting sleeping problems—such as exercise and mindfulness-based stress reduction, may have potential clinical implications for OALWH and their uninfected peers.

Increasing ageism scores were also significantly associated with elevated odds of frailty in OALWH in our study. Ageism, commonly conceptualized as the stereotyping, prejudice and discrimination against people based on age, is becoming increasingly important in older adults [54]. Very few studies have examined the possible association between frailty and ageism. Our finding is consistent with the limited previous research [55]. Persistent exposure to ageism could lead to the internalization of the ageist messages by the OALWH, thus becoming part of their unconscious beliefs. In some cases, the adults may act subconsciously to fulfil the ageist stereotypes, even if detrimental to their health and wellbeing, e.g., physiologic stress response, and physical functioning performance, which may influence frailty. The observed association could also be explained by the health status and higher educational levels among OALWH.

A higher waist/hip ratio was also significantly associated with elevated odds of frailty among OALWH in our study, consistent with previous findings of a positive association between central/abdominal obesity and frailty [56, 57]. As HIV infection has become a manageable chronic illness, it has been progressively accompanied by a growing prevalence of overweight and obesity [58]. Moreover, long-term treatment with cART may contribute to lipodystrophy, often characterized by fat redistribution with a relative increase in abdominal fat [59]. Lipid depositions and infiltration in muscle fibre may bring about frailty by decreasing mobility and increasing loss of muscle strength. Interventions to minimize obesity and sedentary behaviour could potentially be beneficial in addressing frailty.

Visiting traditional healers was significantly associated with elevated odds of frailty among OALWH in our study. We are not aware of previous research that has examined this relationship. Plausibly, frail individuals visit the healers to have their frailty symptoms addressed, especially when the primary care services fail to address these concerns. A previous qualitative exploration of the health and wellbeing of OALWH in the study setting has linked seeking help or treatment from traditional and certain faith healers with poor health outcomes, including defaulting HIV treatment and unsuppressed viral load [60]. Healthcare providers have a reason to be concerned, given the observed impacts of untreated or improperly treated HIV on the development of frailty among OALWH.

Only two HIV-related factors were associated with elevated odds of frailty in our sample: a history of cART regimen change/interruption and prolonged illness following HIV diagnosis. These factors may be indicators of virological failure, ART toxicity or late HIV diagnosis, commonly associated with poorer health outcomes and risk of severe disease. Indeed, previous research has revealed an independent positive association between AIDS diagnosis, viral-load non-suppression, low CD4 count and frailty [17, 61]. Many of the OALWH who have lived with HIV for several years may be significantly impacted by the legacy of the early years of the epidemic, thus predisposing them to a heightened risk of frailty. These adults will more likely require additional support to manage the challenges of ageing with HIV successfully.

Several social factors, e.g., social isolation, social networks, socioeconomic status, social support, social engagement, and social capital, have the potential to influence the health of older adults [62]. In our study, higher monthly household income, residence in a larger household, and having a social network of close friends were all associated with reduced odds of frailty among OALWH, thus confirming previous research [17, 31] and highlighting the importance of promoting positive social factors to aid healthy ageing in older adults living with HIV. These factors may, directly and indirectly, affect frailty, e.g., food security, energy expenditure, and better health-seeking behaviours.

Physical activities have the potential to promote physical function, prevent falls and improve general health, hence delaying the onset and progression of frailty. In our study, participating in light physical activities such as walking was associated with reduced odds of frailty among OALWH, confirming previous research [63]. Exercise may also have positive effects on obesity, stress, loneliness in the case of group activities and muscular strength, which have been identified as potential risk factors for the incidence of frailty. However, light physical activities were associated with elevated odds of frailty among the HIV uninfected older adults in our study. This was a surprise finding, inconsistent with previous investigations [64–66]. This warrants more exploration in the study setting.

Self-reported diabetes was also associated with higher odds of frailty among HIV uninfected older adults in our study, consistent with previous reports [67]. Prospective evidence suggests that unhealthy behaviours and obesity may partly explain the association, and to a larger extent, by poor glucose control and altered serum lipid profile among individuals with diabetes, suggesting that diabetes nutritional therapy may reduce the risk of frailty [67]. Individuals with diabetic neuropathy are particularly at an increased risk of early-onset frailty [68]. Prevention programmes in the pre-frail states through appropriate exercise, nutrition and glycemic control may delay the development of frailty in these adults.

Similar to previous studies, this study showed that males are less likely to be frail than females, suggesting that being male is a protective factor against frailty [24]. Differences in physical activity, muscle mass and higher fat percentages may explain gender discrepancies in frailty. This may also be a question of selection—a classical observation in gerontological research where women live longer but in poorer health [69]. In this respect, men experience more life-threatening chronic conditions compared to women who experience more “non-life-threatening” conditions associated with more morbidity—as such the men who survive are those who tend to have better health status. Still, our observation may be related to social stigma of males appearing/acting/reporting weakness, that is, social preference bias, given the self-reported nature of the construct.

### Implications

The prevalence of frailty in this study was relatively high for both OALWH and their uninfected peers. Preventing, delaying, or treating frailty is more critical in this setting, given its high burden and the fact that frailty is a known predictor of future disability and dependency. Within the clinical practice, an easy-to-use frailty score will allow the easy identification of those at risk, thus allowing planning of future health and social care needs of these adults. Our study also highlighted some of the correlates of frailty in this setting. Many of the factors identified, such as sleeping difficulties, social engagement, ageism, and visiting traditional healers, are potentially modifiable with appropriate programs. Our findings provide the foundation for developing culturally appropriate interventions and healthcare strategies to prevent, delay and manage frailty and its consequences to improve the health and functional status of older adults at risk of frailty. Individual, community-based, or clinic-based interventions such as comprehensive geriatric assessment, physical activity, promoting social engagement, addressing discrimination, and proper management of comorbidities, e.g., diabetes, may benefit older adults. Wider public health approaches, including proactive testing of older adults to avoid late diagnosis and advanced immunosuppression, will benefit OALWH. Our results also highlight the need for well-designed prospective studies to establish the incidence, pathophysiology, predictors of transition and outcomes of frailty in this population and assess potential interventions.

### Strengths and Limitations

Our study is among the very few reports on frailty in SSA and the first one in Kenya. It extends the existing evidence base regarding the prevalence and correlates of frailty in low-resource settings like Kenya. A further strength is the use of a relatively large sample size of people ≥50 years living with HIV and the inclusion of a community-based comparison group which enabled us to give a detailed profile of frailty in this population. We also collected detailed information on sociodemographic, physical, lifestyle and psychological factors, which helped us examine the correlates of frailty in this population. The primary limitation, nonetheless, was the use of a cross-sectional design, which precludes any conclusions on causality. Besides, our sample was predominantly from a rural setting; thus, our participants’ experiences may differ from those in urban places. Also, the OALWH were invited to participate from a health facility (i.e., not a population-based sample) and may not be entirely representative of all OALWH in this area. For instance, the sample of OALWH in the study had very high levels of cART treatment and viral suppression, and our observations would probably be different in individuals not seeking care (either because they are unaware of their status or do not believe they need treatment).

### Conclusion

In this cross-sectional study, we found a significantly higher prevalence of frailty among OALWH (24%) compared to their uninfected peers (13%). However, HIV seropositivity was not significantly associated with frailty after adjustment for demographic, psychosocial and physical factors, underscoring the importance of these factors. Specifically, OALWH who experience sleeping difficulties, ageism, have a high waist/hip ratio, visit traditional healers, have a history of cART regimen change/interruption and prolonged illness after HIV diagnosis have higher odds of frailty. In contrast, those who reside in larger households, have higher household income, have a social network of friends, and engage in light physical activity have reduced odds of frailty. On the other hand, HIV-uninfected older adults with sleeping difficulties, self-reported diabetes and taking part in light physical activities have elevated odds of frailty, while being male is associated with reduced odds of frailty. These factors should be considered in designing and implementing programs to prevent, delay, or treat frailty in this setting. Further prospective work is required to investigate the directionality and potential mediators of the association between frailty and the observed correlates.

## Data Availability

Application for data access can be made through the Data Governance Committee of the KEMRI Wellcome Trust Research Programme who will review the application and advise as appropriate, ensuring that uses are compatible with the consent obtained from participants for data collection. Requests can be sent to the coordinator of the Data Governance Committee using the following email, dgc@kemri-wellcome.org.
